# Tumor Acidosis Boosts Antibody-Driven Cytotoxic Potential of Neutrophils

**DOI:** 10.3390/cells15181663

**Published:** 2026-09-15

**Authors:** Tosca Holtrop, Ida C. van der Peet, Ilona S. T. Hendriks, Tineke Kardol-Hoefnagel, Robin B. Schol, Marta Lustig, Kevin Budding, Jeanette H. W. Leusen, Patricia A. Olofsen, Maria Tsioumpekou

**Affiliations:** 1 Center for Translational Immunology, University Medical Center Utrecht, 3584 CX Utrecht, The Netherlandsi.c.vanderpeet@umcutrecht.nl (I.C.v.d.P.); p.a.olofsen@umcutrecht.nl (P.A.O.); m.tsioumpekou@umcutrecht.nl (M.T.); 2 Division of Stem Cell Transplantation and Immunotherapy, University Medical Center Schleswig-Holstein, 24105 Kiel, Germany

**Keywords:** neutrophils, peripheral blood mononuclear cells (PBMCs), extracellular acidosis, immunotherapy, IgA, IgG, cancer

## Abstract

**Highlights:**

**What are the main findings?**

- Unlike other immune cells, acidity enhances both IgG- and IgA-mediated antibody-dependent tumor cell killing by human neutrophils.
- Acidic pH increases IgA-mediated trogocytosis, LTB4 secretion, and Erk signaling, while reducing ROS production and NET formation.

**What are the implications of the main findings?**

- Neutrophils respond differently from other immune effector cells to extracellular acidity, revealing a distinct regulation of antibody-mediated antitumor activity in the tumor microenvironment.
- These findings highlight the potential of neutrophil-engaging antibody therapies, including IgA-based approaches, for tumors with acidic microenvironments.

**Abstract:**

The acidic tumor microenvironment (pH 6.3–7.1) influences immune cell function, yet its impact on antibody-mediated killing remains poorly understood. While natural killer (NK) cells lose their spontaneous tumor-killing ability in acidic conditions, the effect on IgG-mediated killing is unexplored. Neutrophils, key effector cells in IgA immunotherapy, respond to pH changes by delayed apoptosis and show impaired bacterial killing. In this study, we investigated how extracellular acidity affects immune cell function and modulates antibody-dependent cytotoxicity by neutrophils and peripheral blood mononuclear cells (PBMCs) across multiple in vitro tumor models. Contrary to expectations, acidity significantly enhanced both IgG- and IgA-mediated tumor cell killing by neutrophils, while IgG-mediated antibody-dependent cellular cytotoxicity (ADCC) by PBMCs had a varying impact. This increased neutrophil activity was accompanied by elevated IgA-driven trogocytosis, while protumor reactive oxygen species production and neutrophil extracellular trap (NET) formation were suppressed. Cytokine profiling and Western blot analysis revealed increased Leukotriene B4 (LTB4) secretion and extracellular signal-regulated kinase (Erk) signaling, supporting enhanced neutrophil recruitment and activation in acidic conditions. Our findings reveal that, unlike other immune subsets, neutrophils maintain or even boost antibody-mediated effector functions in the acidic tumor microenvironment, providing new insight into neutrophil biology in tumors and supporting further investigation of neutrophil-targeted antibody immunotherapies.

## 1. Introduction

Cancer cells primarily metabolize glucose via aerobic glycolysis (the Warburg effect [1]), leading to the accumulation of lactate and hydrogen ions in the extracellular environment (pH 6.3–7.1) [2,3]. This acidification promotes tumor proliferation and invasion [4,5,6], while impairing the function of T, NK and dendritic cells by reducing their proliferation, cytokine production and cytotoxicity [7,8,9]. This reduces the effectiveness of immunotherapies such as CAR T cell therapy that rely on robust immune cell activation and persistence within the tumor microenvironment (TME) [10].

Neutrophils are the most abundant leukocyte population in the blood (50–70%) and are emerging as important players in cancer, where they infiltrate the TME and exert both pro- and antitumor functions [11]. Neutrophils can mediate potent antibody-dependent cellular cytotoxicity (ADCC), especially through FcαRI (CD89) engagement of IgA antibodies [12]. This activation is more robust than that induced by IgG, as IgA crosslinking of FcαRI leads to phosphorylation of four immunoreceptor tyrosine-based activation motifs (ITAMs), compared to the two ITAMs activated by FcγRIIIa engagement on NK cells [13].

Neutrophils have been studied under acidic conditions, which revealed an extended life span from 7–19 h [14] to 48 h [15,16]. However, prior work has focused on their roles in inflammation and infection, not on their capacity for antibody-mediated cytotoxicity in cancer. Similarly, studies on NK cells and PBMCs in low-pH environments have only examined spontaneous cytotoxicity. In this study, we address this gap by systematically investigating how extracellular acidification affects both IgG- and IgA-mediated tumor cell killing by neutrophils. To contextualize our findings, we also assess IgG-mediated ADCC by PBMCs under similar conditions.

We demonstrate that, contrary to the broadly suppressive effects of acidic pH on immune function, low pH consistently enhanced IgG- and IgA-induced trogocytosis and tumor cell killing by neutrophils, while IgG-induced PBMC ADCC did not show consistent alterations across varying tumor cell lines. Moreover, cytokine profiling revealed that neutrophils maintain or even enhance their pro-inflammatory responses under acidic conditions, whereas PBMC cytokine secretion is broadly suppressed. Western blot analysis further showed that acidic pH potentiates IgA-mediated Erk1/2 phosphorylation in neutrophils, indicating enhanced activation signaling under low-pH conditions. These findings uncover previously unappreciated functional resilience of neutrophils in the acidic tumor microenvironment and highlight their potential as powerful effectors in antibody-based cancer immunotherapy.

## 2. Materials and Methods

### 2.1. Cell Culture and Cell Lines

Unless stated otherwise, all cell lines were obtained from ATCC and cultured at 37 °C in a humidified incubator containing 5% CO_2_. SKBR3, A431 and UM9-CD38 cells were cultured in RPMI 1640 (Thermo Fisher Scientific, Gibco, Grand Island, NY, USA, Catalog number: 12027599) supplemented with 10% fetal bovine serum (FBS, Sigma-Aldrich, St. Louis, MO, USA, Catalog number: F7524) and 100 U/mL penicillin–streptomycin (Pen/Strep, Gibco, Life Technologies, Waltham, MA, USA, Catalog number: 11528876). The multiple myeloma cell line UM9 was retrovirally transduced with human CD38 to obtain CD38 expression levels comparable to primary myeloma cells and cultured under 0.5 μg/mL puromycin (Sigma-Aldrich, Catalog number: P8833) selection, as described previously [17]. IMR32 cells were maintained in DMEM (Thermo Fisher Scientific, Catalog number: 12077549) supplemented with 10% FBS, 100 U/mL Pen/Strep and 2% MEM Non-Essential Amino Acids Solution (NEAA, Gibco, Life Technologies, Catalog number: 12084947). Cells were not cultured past 30 passages and were regularly tested for mycoplasma contamination using a Mycoalert mycoplasma detection kit (Lonza, Basel, Switzerland, Catalog number: LT07-318).

### 2.2. Antibodies and Reagents

IgG antibodies against human epidermal growth factor receptor 2 (HER2, Herceptin/Trastuzumab, Roche, Basel, Switzerland), epidermal growth factor receptor (EGFR, Cetuximab, Merck, Rahway, NJ, USA), and CD38 (Daratumumab, Janssen, Beerse, Belgium) were obtained via the pharmacy of UMC Utrecht. The disialoganglioside (GD2) antibody (Dinutuximab/IgG1 ch14.18) was produced by WuXi Biologics (Wuxi, China) (RRID:SCR_001217) in CHO-K1 cells and subsequently purified. The IgA3.0 antibodies, henceforth called IgA, against HER2 (Trastuzumab), EGFR (Cetuximab), and GD2 (ch14.18) were produced and purified in-house following previously described methods [18]. The IgA2.0-CD38 was kindly gifted by Prof. Dr. Thomas Valerius. An overview of all antibodies used for flowcytometry, immunohistochemistry and immunoblot staining can be found in Table 1.

Acidified culture medium (pH 6.3) was prepared by adding 1.5–2 mL/L lactic acid (Honeywell Fluka, Seelze, Germany, Catalog number: 5636730) to RPMI with low HEPES, low bicarbonate and no glutamine (Gibco, Catalog number: 42402016) to minimize buffering of the pH.

### 2.3. PBMC/Neutrophil Isolation and Overnight Incubation

Peripheral blood was collected from healthy donors at UMC Utrecht. Human polymorphonuclear leukocytes (PMNs, mainly comprising neutrophils and therefore hereafter referred to as neutrophils) and PBMCs were isolated via Ficoll (VWR, Radnor, PA, USA, Catalog number: 17-1440-03) density gradient centrifugation. PBMCs were isolated from the middle layer, leaving only red blood cells (RBCs) and neutrophils in the pellet. RBCs were lysed with RBC Lysis Buffer (Biolegend, San Diego, CA, USA, Catalog number: 420302) and neutrophils were used for ADCC assays.

For most assays, cells were used immediately, exposing effector cells to acidic pH for 4 h. In the 24 h assays, neutrophils or PBMCs were resuspended in normal or acidic RPMI medium (up to 2 million cells/mL) and transferred to a T75 cell culture flask for adequate CO_2_ exchange. To the neutrophils, 100 U/mL of granulocyte colony-stimulating factor (i.e., CSF3/G-CSF, Immunotools, Friesoythe, Germany, Catalog number: 11343133) was added. The cells were then cultured for 20 h at 37 °C with 5% CO_2_, followed by 2 or 4 h assays (based on the assay) at their respective pH.

### 2.4. ^51^Cr Release ADCC Assay

ADCC assays were performed as described previously [19]. In short, target cells were labeled with 100 µCi chromium-51 (^51^Cr, Revvity, Waltham, MA, USA, Catalog number: NEZ030S001MC) per million cells and incubated for at least 2 h at 37 °C and 5% CO_2_. Subsequently, cells were washed three times with medium.

Freshly isolated neutrophils were combined with the target cells at a 40:1 effector-to-target (E:T) ratio and PBMCs at a 100:1 E:T ratio, along with antibodies at concentrations specified in the experiment in RPMI medium with either a normal or acidic pH. Following a 4 h incubation at 37 °C in a humidified incubator containing 5% CO_2_, the plate underwent centrifugation, and the supernatant was transferred to a LumaPlate (Revvity, Catalog number: 6006633) for measurement on a beta-gamma counter (PerkinElmer, Shelton, CT, USA) to determine radioactive scintillation (in counts per minute). Specific lysis was computed using the following formula: ((experimental cpm − basal cpm)/(maximal cpm − basal cpm)) × 100. The maximal cpm was established by treating target cells with 5% Triton X-100 (Sigma-Aldrich), while the baseline cpm was determined by chromium release from target cells without antibodies and effector cells.

### 2.5. Flow Cytometry Assay

Flow cytometry was used to determine changes in viability, Fc receptor expression, and immune cell composition of isolated PBMC and neutrophil fractions. For all flow cytometric assays, 1 × 10^5^ cells per well/tube were seeded and incubated with the staining antibodies (listed in Table 1) in FACS buffer (PBS, 0.01% BSA, 0.01% Na-Azide) for 45 min at 4 °C, after which the cells were washed with PBS and resuspended in FACS buffer before measuring on a BD FACSCanto II (BD Biosciences, Franklin Lakes, NJ, USA). For viability assays, cells were resuspended in Annexin binding buffer after antibody staining. Annexin V and 7-AAD were added to identify apoptotic and viable cells, respectively. Following a 15 min incubation at RT in the dark, samples were diluted further in Annexin binding buffer and analyzed by flow cytometry within 1 h. Flow cytometric analysis was performed using FlowJo v10.8.1 software. To assess the immune cell composition of the isolated PBMCs and neutrophil fractions, cells were stained using antibodies against CD66b, CD14, CD16, CD56, CD3, CD20, and CD19. NK cells were defined as CD16^+^CD56^+^, neutrophils as CD66b^+^, NKT-like cells as CD3^+^CD56^+^ and T and B cells as CD3^+^ and CD19^+^CD20^+^ respectively, while monocyte subsets were identified based on their CD14 and CD16 expression. Fc receptor expression on NK cells and neutrophils was evaluated using antibodies against CD16, CD32, CD64 and CD89. For gating strategies, see Figures S2 and S3.

### 2.6. Trogocytosis

SKBR3 cells were labeled with 5 μM DiO (Thermo Fisher Scientific, Catalog number: 10023132) according to the manufacturer’s instructions. The labeled cells were co-cultured with neutrophils at an E:T ratio of 5:1 in complete RPMI for up to 4 h at 37 °C in a humidified incubator containing 5% CO_2_, with 10 μg/mL IgG or IgA against HER2, GD2 or EGFR at either pH 6.3 or pH 7.4. After co-culture, cells were washed with PBS and neutrophils were stained with anti-CD66b-V450. The frequency of DiO^+^ tumor cells taken up by CD66b^+^ neutrophils was measured by flow cytometry using a BD FACS Canto II flow cytometer.

### 2.7. Fc Binding Assays

For binding assays performed in 96-well flat-bottom NUNC Maxisorp plates (Thermo Fisher Scientific, Catalog number: 439454), wells were coated overnight at 4 °C with 1 or 10 μg/mL antibody (IgA or IgG against EGFR, GD2, HER2 or CD38) in carbonate–bicarbonate buffer (Sigma-Aldrich, Catalog number: C3041-50CAP). After coating, plates were washed with PBS three times and blocked with 1% BSA in PBS for an hour at RT.

Isolated neutrophils were labeled with 20 μM Calcein-AM (Thermo Fisher Scientific, Catalog number: 10462052) for 30 min at 37 °C, washed twice with complete medium, and resuspended in normal or acidic pH medium. A total of 1.5 × 10^5^ calcein-labeled neutrophils in 100 µL per well were subsequently added to the antibody-coated plates. The plates were gently centrifuged for 5 min at 300 rpm and incubated for 30–45 min at 37 °C with 5% CO_2_. Following incubation, cells were washed 10 times with the normal or acidic pH medium. Fluorescence was measured every two washes using a Spectramax microplate reader (Molecular Devices, Silicon Valley, CA, USA, excitation: 485 nm, emission: 527 nm), and the procedure was repeated until a total of 10 washes were completed.

### 2.8. Superoxide Production

Superoxide production was measured on 4 × 10^5^ freshly isolated neutrophils and after 20 h incubation. IgG-HER2 or IgA3.0-HER2 antibodies were added at a concentration of 10 µg/mL, together with 1 × 10^4^ SKBR3 cells. Then, 25 ng/mL phorbol myristate acetate (PMA, Sigma-Aldrich, Catalog number: P8139) was used as positive control. After a 3 min centrifugation at 40 *g* and 4 °C, 150 µM luminol (Sigma-Aldrich, Catalog number: 521-31-3) was added. The plate was placed in a luminometer (SpectraMax M3, Molecular Devices) set at 37 °C, after which the chemiluminescent reaction was measured as function of time. The photoemission in relative light units (RLU) was measured every 66 s for 60 min.

### 2.9. NETosis ELISA and Immunofluorescence Microscopy

Neutrophils were isolated and either used directly or kept overnight in either pH 7.4 or pH 6.3. In a 24-well plate, containing round glass coverslips, 1 × 10^6^ neutrophils were incubated together with 2.5 × 10^4^ SKBR3 cells in the presence or absence of 10 µg/mL IgG-HER2 or IgA-HER2 at 37 °C and 5% CO_2_. After 4 h, supernatant was collected, and cells were fixed on coverslips with 4% paraformaldehyde (PFA, VWR, Catalog number: VWRK4078-9001) for 10 min.

For immunofluorescence, cells were permeabilized and blocked using 5% BSA + 0.05% Triton X-100 (Sigma-Aldrich) for 30 min, after which NETs were stained overnight at 4 °C with Anti-Myeloperoxidase-FITC (MPO-FITC) (Agilent, Santa Clara, CA, USA, dilution 1:100). Coverslips were mounted onto slides with VECTASHIELD^®^ PLUS Antifade Mounting Medium with DAPI (Vector Laboratories, Newark, CA, USA, Catalog number: H-1900-2) and sealed with clear nail polish. Subsequent imaging was performed using the Thunder RT widefield microscope (GE Healthcare, Chicago, IL, USA) at 20× magnification.

To quantify circulating NETs, anti-MPO antibody (Bio-Rad, Hercules, CA, USA) was coated overnight at 5 μg/mL on a 96-well Maxisorp plate at 4 °C. The plate was then washed with PBS-Tween 0.05% (PBST) and blocked using PBST + 1%BSA. Supernatant was diluted 50-fold in PBS and applied to the plate for 2 h at RT. After washing, anti-DNA-POD (from Cell Death Detection enzyme-linked immunosorbent assay (ELISA) kit, Sigma-Aldrich, Catalog number: 11544675001) was diluted according to manufacturer’s instructions and added for 1.5 h at RT and subsequently detected with ABTS solution (ITK Diagnostics, Uithoorn, The Netherlands, Catalog number: AAT 11001). Absorbance of the plate was measured using the Spectramax microplate reader (Thermo Fischer Scientific) at a wavelength of 405 nm.

### 2.10. Multiplex Cytokine Assay

A multiplex bead-based assay panel was used to determine the section of various cytokines by freshly isolated or 20 h cultured neutrophils and PBMCs in normal and acidic pH. Supernatant was obtained in a similar set-up as the ^51^Cr release assays with the exception of adding the radioactive chromium, and included an incubation of 4 h. The COVID-19 Cytokine storm Panel 1 (13-plex, Biolegend, Catalog number: 741091) was used, which can be used to quantify 13 human cytokines: IL-6, MCP-1 (CCL2), G-CSF, IFN-α2, IL-2, IFN-γ, IL-7, IL-1RA, IL-8 (CXCL8), TNF-α, IP-10 (CXCL10), MIP-1α (CCL3) and IL-10. Then, 25 µL supernatant was used following the manufacturer’s instructions and measured on a BDCanto II.

### 2.11. LTB4 Measurements

LTB4 levels in supernatant samples (same set-up as ^51^Cr release assays but without the chromium) were determined by a commercial ELISA kit according to the manufacturer’s protocol (Enzo Life Sciences, Long Island, NY, USA, Catalog number: 89141-118). The concentrations of LTB4 were obtained from standard curves of positive control proteins from the kits with a four-parameter logistic regression model. When the concentration of LTB4 was lower than 5.63 pg/mL (the lowest limit of the standard curve), the value was set as equal to zero, and when it was more than 3000 pg/mL (the highest limit of the standard curve), the value was set to 3000.

### 2.12. Live-Cell Imaging

SKBR3 cells were stained with 1 µM CellTrace carboxyfluorescein succinimidyl ester (CFSE, Thermo Fisher, Catalog number: 15530597) according to the manufacturer’s instructions and incubated for 2 h at 37 °C and 5% CO_2_ in an eight-well µ-slide (Ibidi, Gräfelfing, Germany, Catalog number: 80824). Neutrophils were isolated and stained with Cell Trace Violet (CTV, Thermo Fisher Scientific, Catalog number: C34557) according to the manufacturer’s instructions. Following this, 10 µg/mL of IgG-HER2 or IgA-HER2 was added to the target cells. After an incubation period of 30 min, neutrophils (at the corresponding pH) were introduced, together with 1 µg/mL propidium iodide (Merck), at an effector:target cell ratio of 10:1. The cells were maintained in a controlled environment at 37 °C and 5% CO_2_ within an enclosed incubation chamber on a Thunder RT widefield microscope (GE Healthcare). This set-up included a 40× camera. Imaging was conducted at 27 s intervals for a duration of 2 h. Neutrophil roundness was determined using the SAMJ plugin in FIJI 1.53n (https://arxiv.org/abs/2506.02783, last accessed on 11 June 2025). In short, the EfficientViTSAM-l2 model of the SAMJ plugin was loaded in FIJI, after which neutrophils were manually selected using the selection tool. When all regions of interest were selected, shape descriptors, including roundness, were determined by clicking analyze. The neutrophil speed (mean track displacement in µm/minute) and displacement (mean square displacement in µm^2^) were tracked automatically using the Trackmate module in FIJI [20] and subsequently analyzed using the MotilityLab website (http://www.motilitylab.net/, last accessed on 17 December 2024).

### 2.13. Immunoblot

Neutrophils were isolated and resuspended in Hanks’ buffer (HyClone, Logan, UT, USA, Catalog number: SH30268.01) at a concentration of 1 × 10^8^ cells/mL. Her2-IgA and Her2-IgG antibody-coated beads were prepared at 1 × 10^8^ beads/mL, as described elsewhere [13]. For each experimental condition, neutrophils were stimulated with beads at a 1:4 neutrophil-to-bead ratio at 37 °C for the indicated timepoints. Following stimulation, cells were lysed by boiling in 4× Laemmli buffer (Bio-Rad, Catalog number: 1610747) containing DTT for 10 min at 95 °C. Samples were then centrifugated at maximum speed for 5 min, and the supernatants were collected and stored at −20 °C until further analysis.

Proteins were separated by 12% SDS-PAGE gels (Bio-Rad, Catalog number: 4561044) and transferred onto nitrocellulose membranes using the Trans-Blot Turbo Transfer System (Bio-Rad; mini gel program). Membranes were blocked for 1 h at room temperature (RT) in 5% BSA in TBS containing 0.1% Tween-20 (TBST) and incubated overnight at 4 °C with primary antibodies (p-Erk1/2). The following day, membranes were washed three times with TBS-T and incubated for 1 h at RT with infrared dye 800cw anti-rabbit IgG antibodies in 1% BSA in TBS-T. Protein bands were visualized by Odyssey Sa imaging (Li-Cor, Lincoln, NE, USA; intensity 8; resolution 100 μm; focus 3 mm).

For β-actin detection, membranes were washed in TBS-T, and subsequently blocked, washed and re-probed with primary and secondary (infrared dye 680rd anti-mouse IgG) antibodies and visualized by Odyssey Sa imaging. Image Studio v5.2.5 software (Li-Cor) was used for analysis and quantification.

### 2.14. Data Processing and Statistical Analysis

Flow cytometry analysis was performed with BD FACSDiva (BD Biosciences) and FlowJo v10.8.1 software (FlowJo, Ashland, OR, USA). Statistical analysis was done using GraphPad Prism 10.1.2 (GraphPad Software Inc., Boston, MA, USA). Specific statistical tests performed for each experiment are indicated in the corresponding figure legends. Data is presented as mean ± standard error of the mean (SEM), where a *p*-value < 0.05 was considered a significant difference. Graphs and figures were generated using the aforementioned software and Adobe Illustrator. Imaging data was analyzed using Imaris (Bitplane, Zürich, Switzerland).

## 3. Results

### 3.1. Enhanced Antibody-Mediated Lysis with Both IgG and IgA in Acidic Environment

**Figure 1:**
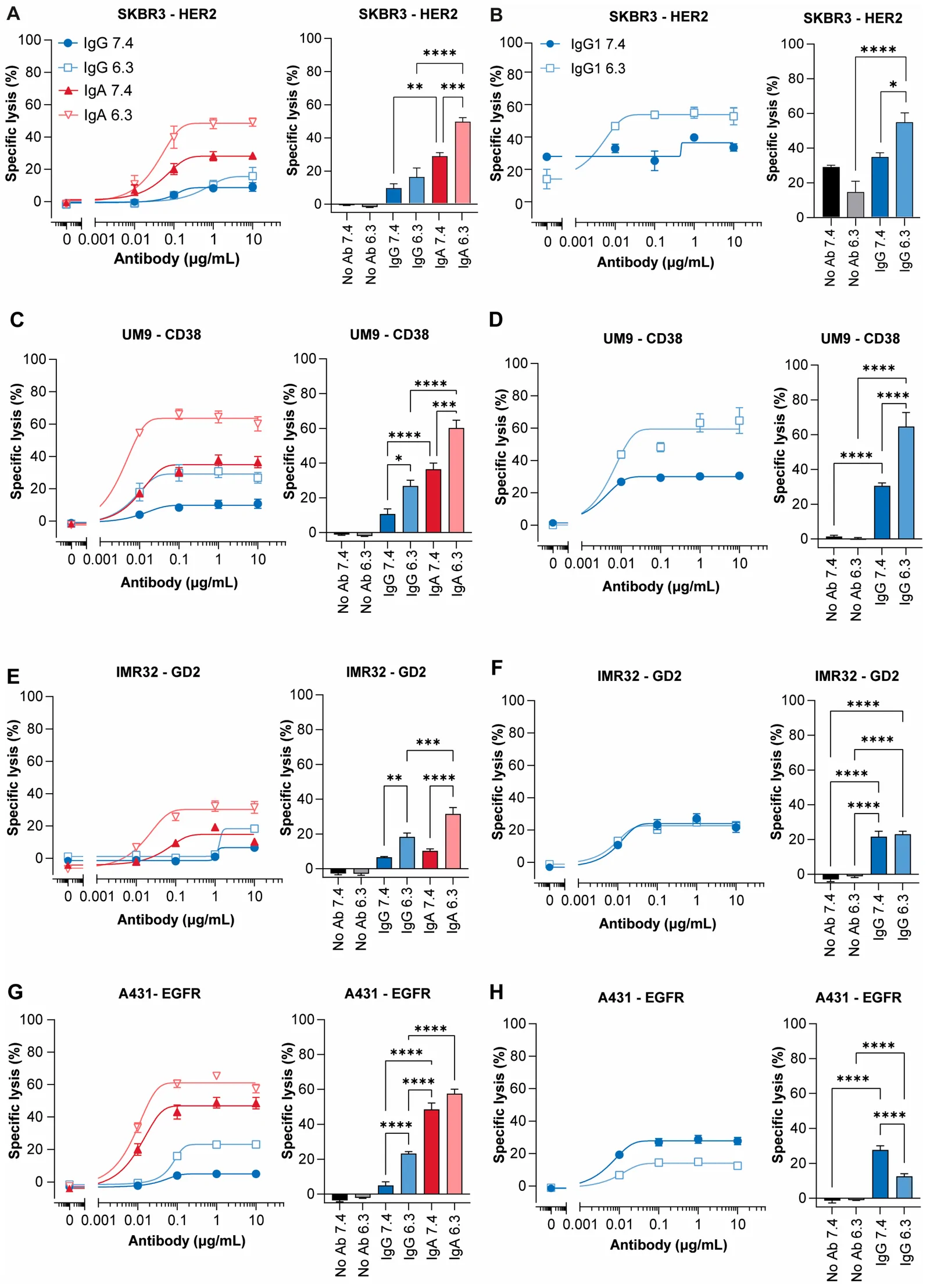
Antibody-mediated lysis of four different tumor cell lines with neutrophils or PBMCs at pH 7.4 and pH 6.3. (**A**,**C**,**E**,**G**) Specific tumor lysis by freshly isolated neutrophils (E:T = 40:1) or (**B**,**D**,**F**,**H**) PBMCs (E:T = 100:1) using a 4 h ^51^Cr release assay at pH 7.4 or 6.3. The IgG and IgA formats of (**A**,**B**) Trastuzumab (HER2) to target SKBR3 cells, (**C**,**D**) Daratumumab (CD38) to target UM9 cells, (**E**,**F**) Dinutuximab (ch14.18/GD2) to target IMR32 cells, and (**G**,**H**) Cetuximab (EGFR) to target A431 cells were used. Mean with SEM is shown for *n* = 3–4 independent experiments with different healthy donors. Bar graphs show mean specific lysis at 10 μg/mL for IgG and IgA antibodies of 3–4 experiments. No Ab indicates condition without antibody. * *p* < 0.05, ** *p* < 0.01, *** *p* < 0.001, **** *p* < 0.0001, one-way ANOVA with Tukey’s post hoc test.

Killing of the human epidermal growth factor receptor 2 (HER2)-positive breast cancer cell line SKBR3 with IgG- and IgA-Trastuzumab was assessed at neutral and acidic pH using both neutrophils and PBMCs. IgG-mediated killing by neutrophils remained low and unchanged at acidic pH (Figure 1A), whereas IgA induced a strong 1.7-fold increase in tumor cell killing under acidic conditions. As expected, the spontaneous tumor lysis with PBMCs was reduced at acidic pH (Figure 1B), yet antibody-mediated tumor lysis was significantly enhanced at low pH (1.6-fold increase). These results indicate that extracellular acidosis positively impacts antibody-mediated lysis of SKBR3 cells with both neutrophils and PBMCs.

Next, we examined whether antibody-mediated tumor lysis remained possible, and could potentially be enhanced, following prolonged exposure to acidic pH. This was of particular interest given previous reports that NK cells lose their spontaneous tumor-killing ability after 20 h, an effect that is not reversible even upon subsequent culture at pH 7.4 [9]. Hence, we cultured purified NK cells, PBMCs and neutrophils for 20 h at pH 7.4 or 6.3 and then used them in a 4 h ADCC experiment at their respective pH. As expected, after 24 h at acidic pH, NK cells lost their spontaneous cytotoxicity (2.7% lysis; Figure S1A), while activity increased at neutral pH (13% after 4 h to 33.8% after 24 h, Figure S1B). Interestingly, NK cells retained potent IgG-mediated tumor killing after prolonged exposure to acidic pH, with lysis comparable to neutral pH (~60%). After 24 h, IgG-mediated lysis by PBMCs at pH 7.4 increased by nearly 20%, whereas lysis remained similar at pH 6.3 (48% at 4H versus 51% at 24H, Figure S1C), resulting in no significant differences between pH levels at 24H. In contrast, neutrophils showed a 10–20% reduction in overall antibody-mediated lysis after 24 h at both pH conditions but remained markedly higher under acidic conditions (Figure S1D). Together, the preserved IgG-mediated tumor killing by purified NK cells under acidic conditions supports that NK cells are the main contributor to the antibody-mediated effects observed with PBMCs.

To assess whether the increased lysis at acidic pH is tumor- or tumor-associated-antigen-specific, we tested three additional cell lines: UM9-CD38 (multiple myeloma; CD38), IMR32 (neuroblastoma; disialoganglioside GD2), and A431 (epidermoid carcinoma; epidermal growth factor receptor, EGFR). IgG-mediated ADCC was significantly enhanced in acidic pH in UM9 (2.5-fold, Figure 1C), IMR32 (2.8-fold, Figure 1E), and A431 cells (4.6-fold, Figure 1G). IgA-mediated lysis also increased under acidic conditions for UM9 (1.6-fold) and IMR32 (3.1-fold), albeit not significantly for A431 cells (1.2-fold, *p* = 0.07). PBMCs effectively mediated IgG-dependent lysis of all cell lines at either pH (Figure 1D,F,H). However, enhanced lysis at acidic pH was observed only for UM9 (2.1-fold), while IMR32 showed consistent lysis regardless of pH. In A431, lysis was significantly reduced under acidic conditions. Given these findings, we focused primarily on neutrophils in subsequent experiments, and PBMC data, if available, is provided in the Supplementary Materials for reference.

### 3.2. Acidic pH Enhances Mainly IgA-Mediated Trogocytosis

Neutrophils can also mediate ADCC of antibody-opsonized tumor cells through trogoptosis, a mechanism that targets and destroys the cancer cell membranes via trogocytosis [21]. We investigated the sensitivity of A431, SKBR3, and IMR32 cell lines to pH-dependent neutrophil-mediated trogoptosis.

**Figure 2:**
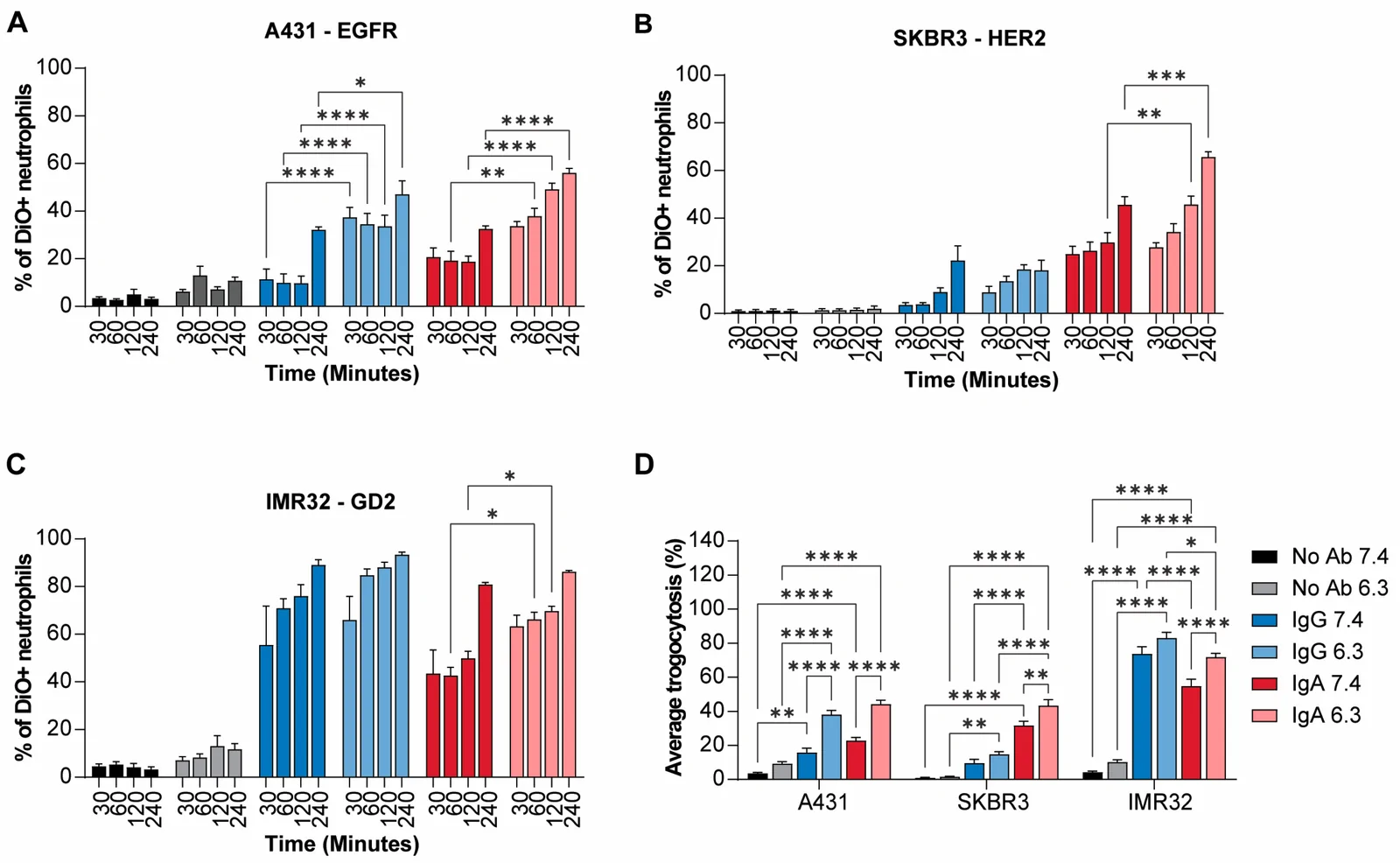
Increased trogocytosis observed in acidic pH for all cell lines tested. (**A**) A431, (**B**) SKBR3 and (**C**) IMR32 cells were labeled with DiO, a fluorescent lipophilic probe that gets incorporated into the plasma membrane. Antibody-mediated trogocytosis was quantified by measuring the percentage of DiO^+^ neutrophils over time. (**D**) Bar graph of mean antibody-mediated trogocytosis per cell type. Two-way ANOVA with Tukey multiple comparison test. *n* = 3, in duplicate. * *p* = 0.05, ** *p* < 0.01, *** *p* < 0.001, **** *p* < 0.0001.

Acidic pH significantly enhanced the average IgG- and IgA-mediated trogocytosis of A431 cells by 2.4-fold and 2.0-fold, respectively (Figure 2A,D). In SKBR3 cells, IgG-mediated trogocytosis was not significantly affected by acidic conditions, whereas IgA-mediated trogocytosis increased by 1.4-fold (Figure 2B,D). This increase in trogocytosis cannot be attributed to phagocytosis of tumor apoptotic material since apoptotic levels are low and do not alter upon acidosis (Figure S2A). In IMR32 cells, IgG-mediated trogocytosis showed a modest, non-significant increase (1.1-fold, *p* = 0.06), while IgA-mediated trogocytosis was significantly elevated by 1.3-fold (Figure 2C,D). In conclusion, acidic pH significantly enhances antibody-mediated trogocytosis in a cell type-dependent manner. The effect was most pronounced for A431 and IMR32 cells, particularly with IgA, whereas SKBR3 cells showed minimal sensitivity to pH changes on IgG-mediated trogocytosis, indicating that the impact of pH on this process may vary across different tumor cell lines.

### 3.3. Extracellular Acidosis Influences Antibody Binding, ROS Production and NETosis of Neutrophils

Numerous studies have shown that an acidic TME affects immune cell viability, surface marker expression, and function [7,8,9]. We observed no significant changes in acidic conditions in immune cell composition within the PBMC and neutrophil fraction, as well as surface marker expression (Figures S2 and S3). However, a significantly improved neutrophil viability was observed after 20H in acidic pH (Figure S2E). Hence, these changes cannot explain the increased neutrophil-mediated tumor lysis observed after 4 h but probably contribute to the differences observed after 24 h.

**Figure 3:**
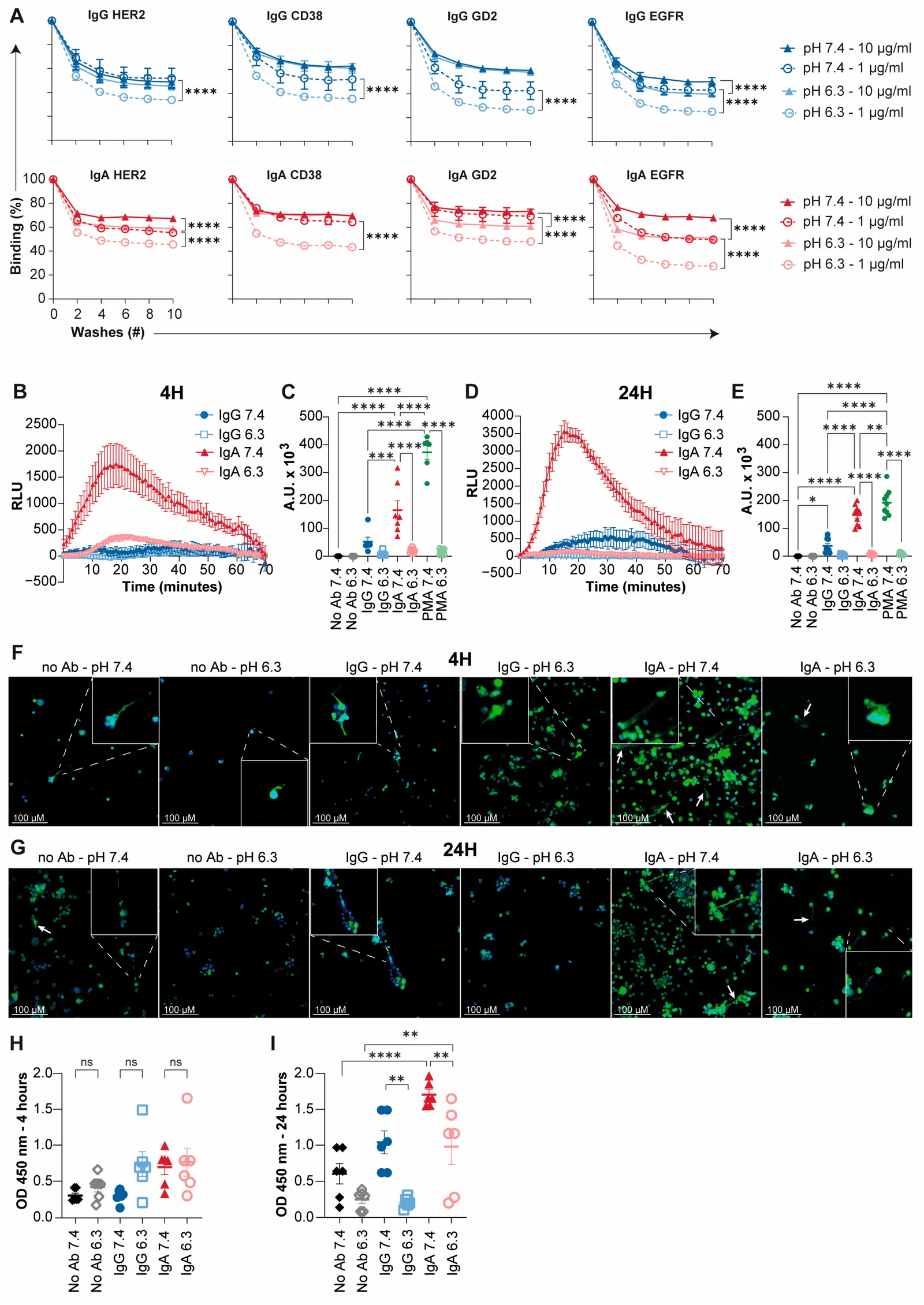
Extracellular acidosis influences antibody binding, ROS production and NETosis in neutrophils. (**A**) Binding curves of neutrophils to IgG- and IgA-HER2, -CD38, -GD2 and –EGFR-coated plates, with 10 washing rounds. Significance was determined based on average binding of all washes per pH. Mean values ± SEM (% cells) from three/four independent experiments for each timepoint and pH are given. Two-way ANOVA with Tukey’s post hoc test. * *p* < 0.05, *** *p* < 0.001, **** *p* < 0.0001. (**B**–**E**) Superoxide burst in relative light units (RLU) with (**B**,**C**) freshly isolated neutrophils or (**D**,**E**) upon overnight culture at their respective pH. Lines show mean of *n* = 3—6 measurements, in triplicate. (**C**,**E**) AUC of superoxide burst by neutrophils induced by IgG or IgA antibodies, including PMA as positive control. (**F**–**I**) NETosis measurement in neutrophils after 4 and 24 h. Immunofluorescent images of NETs after (**F**) 4 and (**G**) 24 h. NETs were stained with anti-MPO FITC overnight, after which DAPI was added. NETs are highlighted by magnified insets in the corners and by white arrows. (**H**,**I**) NET ELISA on supernatant of microscope samples. NETs were quantified with anti-MPO antibody and anti-DNA-POD antibody. Ordinary one-way ANOVA with Tukey multiple comparison test. *n* = 3, in duplicate. ns = not significant, * *p* = 0.05, ** *p* < 0.01, *** *p* < 0.001, **** *p* < 0.0001. AUC = area under the curve, RLU = relative light unit.

We next assessed whether extracellular pH influences antibody binding to neutrophils, which is critical for downstream effector functions. Using IgG and IgA antibodies against HER2, CD38, GD2, and EGFR, we observed significantly reduced binding at pH 6.3 for all antibodies at 1 µg/mL (*p* < 0.0001, Figure 3A). At 10 µg/mL, IgG binding was largely maintained across pH conditions, except for IgG-EGFR, which showed reduced interaction at low pH. In contrast, IgA binding remained reduced for all targets (*p* < 0.0001), except CD38. Notably, despite the decrease in binding, antibody-mediated cytotoxicity was not impaired.

To investigate whether acidic pH influences other neutrophil effector functions, we examined reactive oxygen species (ROS) production and neutrophil extracellular trap (NET) formation as it is reported that acidic pH suppresses PMA-induced ROS and subsequent NETosis [15]. Using a luminol-based assay, we found that IgA induced strong ROS production at pH 7.4, whereas IgG triggered only a modest response (Figure 3B,C). At pH 6.3, neither IgA, IgG, nor PMA induced detectable ROS, even after 20 h (Figure 3D,E), indicating complete suppression of superoxide generation under acidic conditions. In addition, detectable NET formation was observed after 4 h in the presence of IgA in both pH conditions compared to the baseline levels observed in the absence of antibody (Figure 3F,H). However, 24 h IgA, and to a lesser extend IgG, induced significant NET formation at both neutral and acidic pH, though levels were significantly lower in acidic pH (Figure 3F,I).

### 3.4. Acidic pH Alters Neutrophil Morphology and Behavior

Having established that acidic pH does not impair, but rather enhances, ADCC, we next sought to visualize how pH influences neutrophil behavior during ADCC in real time. Using a live-cell-imaging ADCC assay with a low effector–target ratio (10:1), we tracked tumor cell killing kinetics and neutrophil behaviors such as displacement, speed and morphological changes in response to IgG, IgA, and pH.

**Figure 4:**
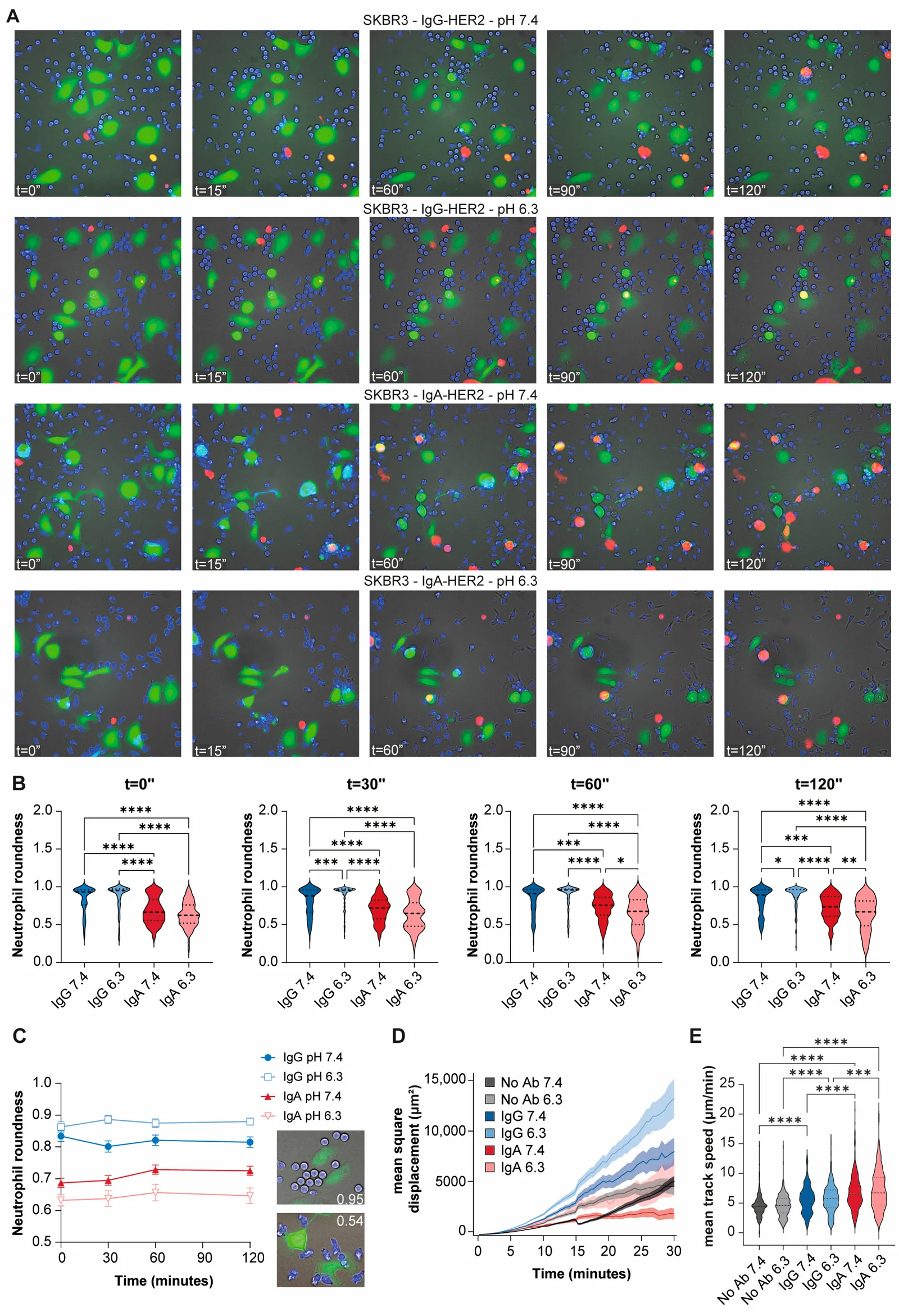
Effect of pH on neutrophil behavior and morphology. (**A**) Live imaging ADCC of SKBR3 cells with neutrophils (E:T = 10:1), monitoring killing over 120 min. SKBR3 cells were labeled with CFSE (green) and neutrophils with CTV (blue). Cell death is expressed as the percentage of propidium iodide-positive (red) tumor cells relative to t = 0 and is displayed in the upper right corner. (**B**,**C**) Neutrophil roundness over time in response to IgG or IgA in neutral or acidic pH. Examples of round non-activated (upper image) vs. spread-out activated (bottom image) neutrophils and their equivalent mean roundness. Significance determined with two-way ANOVA with Bonferroni post hoc test. * *p* = 0.05, ** *p* < 0.01, *** *p* < 0.001, **** *p* < 0.0001. (**D**,**E**) Tracking of neutrophils with SKBR3 cells (E:T = 10:1) with IgG- and IgA-HER2 antibodies in live imaging over 30 min. (**D**) Directional movement of neutrophils in mean square displacement (i.e., deviation from a cell’s starting point over 30 min) and (**E**) migration velocity in mean track speed (i.e., mean distance a cell travels in µm in 1 min). Kruskal–Wallis test with Dunn’s multiple comparisons. *** *p* < 0.001, **** *p* < 0.0001.

Real-time recordings revealed enhanced neutrophil swarming in IgA compared with IgG conditions, which resulted in increased neutrophil-mediated killing. This effect was visualized by the conversion of SKBR3 cells from green to red fluorescence (Figure 4A, Videos S1–S6). This suggests that IgA more effectively drives neutrophil recruitment and clustering around tumor targets, regardless of pH.

To further assess neutrophil activation upon antibody treatment and pH changes, we analyzed cell roundness. Across all timepoints, neutrophils stimulated with IgA were significantly less round than those exposed to IgG, indicating greater spreading and activation (Figure 4B,C). This morphological difference was already evident at baseline (mean roundness: IgA ~0.65 vs. IgG ~0.85). Acidic pH had isotype-specific effects: IgA-treated neutrophils maintained an activated, spread-out morphology across all timepoints, whereas in IgG-treated conditions, acidity reduced roundness at 30 and 120 min (Figure 4B,C). Nonetheless, neutrophils remained consistently more round with IgG, emphasizing the stronger activation state induced by IgA.

Lastly, we tracked neutrophil motility in response to antibody treatment using two metrics: directional movement (mean square displacement) and migration velocity (mean track speed). Acidic pH enhanced directional movement with either IgG or IgA (Figure 4D and Figure S4). IgG induced the furthest displacement, characterized by long, linear tracks (Figure S4), while IgA-treated cells showed localized migration and clustering around tumor cells. No-antibody controls exhibited limited movement with short, random paths. Track speed was not affected by pH within antibody conditions (Figure 4E). However, both IgG and IgA significantly increased neutrophil speed at either pH and speeds were notably higher with IgA.

### 3.5. IgA Triggers LTB4 Release and Enhances Erk1/2 Signaling at Acidic pH

To better understand the functional impact that pH alterations have on neutrophils during ADCC, we analyzed cytokine and chemokine release following IgG- or IgA-opsonized SKBR3 exposure under normal and acidic pH using a multiplex assay.

**Figure 5:**
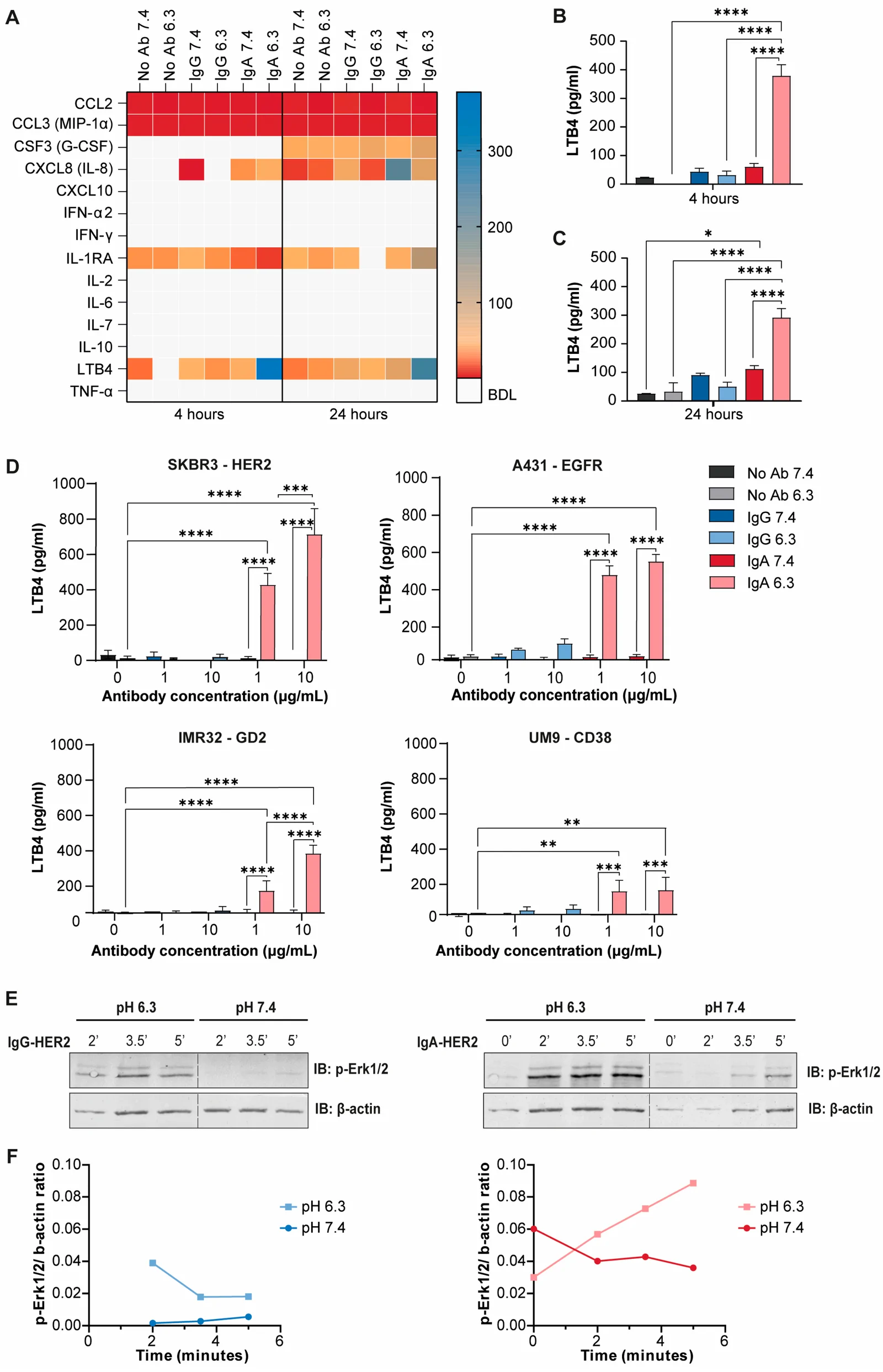
Acidic pH enhances LTB4 secretion and Erk1/2 signaling, particularly upon IgA treatment. (**A**) Multiplex cytokine array of SKBR3 cells with neutrophils (IgG and IgA) in normal or acidic pH. Immune cells were freshly isolated from 3 healthy donors and either directly used in the assay for 4 h or cultured overnight in medium at their respective pH. G-CSF was added to the medium to ensure neutrophil viability. Concentration in pg/mL. BDL = below detection limit. (**B**,**C**) Bar graphs of LTB4 excreted by neutrophils as detected with the multiplex assay after (**B**) 4 h and (**C**) 24 h. (**D**) LTB4 ELISAs of SKBR3, A431**,** IMR32, and UM9 cells, co-cultured with neutrophils in the presence or absence of antibodies in normal or acidic pH. The same set-up was used as with the multiplex cytokine array, but only 4 h timepoint and 3 different healthy donors were used, measured in duplicate. Mean + SEM, two-way ANOVA, and Tukey’s post hoc test. * *p* < 0.05, ** *p* < 0.01, *** *p* < 0.001, **** *p* < 0.0001. (**E**) Representative Western blots and subsequent quantification (**F**) showing phosphorylated Erk1/2 (p-Erk1/2) and actin in neutrophils cultured under normal or acidic pH for the indicated timepoints following IgA-HER2 or IgG-HER2 stimulation, *n* = 2.

Of the 14 analytes measured, only Leukotriene B4 (LTB4), a potent neutrophil chemoattractant and activator, was highly responsive to IgA in acidic pH after 4 and 24 h (Figure 5A–C). An increase in LTB4 secretion could be observed in neutral pH as well but only reached significance after 24 h (Figure 5C). In contrast, PBMCs showed a distinct pattern: IgG antibody treatment induced C-C motif chemokine ligand 2 (CCL2/MCP-1), C-X-C motif ligand 8 (CXCL8/IL-8), and interleukin-1 receptor alpha (IL-1RA) secretion at pH 7.4 after 4 h, but all responses were fully suppressed under acidic conditions. By 24 h, only CCL2 remained elevated with IgG at normal pH (Figure S5A–D).

To further explore LTB4 signaling, we tested three additional tumor lines via ELISA. In the absence of antibody, LTB4 levels were negligible (Figure 5D). IgA consistently triggered a dose-dependent LTB4 release at low pH across all models, whereas IgG elicited only limited and inconsistent LTB4 release in PBMCs (Figure S5E). However, IgA stimulation consistently induced LTB4 release by neutrophils across all tumor models, but only under acidic conditions, highlighting their ability to remain functionally active in acidic microenvironments. Moreover, increasing the IgA concentration from 1 to 10 µg/mL further enhanced LTB4 secretion in both SKBR3 and IMR32 co-cultures. In contrast, PBMCs showed a much more limited LTB4 response (Figure S5E).

While IgG at acidic pH triggered some LTB4 production in response to A431 and IMR32, this effect was not observed as consistently across cell lines or conditions. Consistent with our cytokine and ELISA results, immunoblot analysis revealed enhanced neutrophil activation, particularly increased Erk1/2 phosphorylation, under acidic compared to normal pH conditions (Figure 5E,F). IgG treatment induced a modest rise in p-Erk1/2 levels, whereas IgA elicited a more robust and sustained activation. These findings indicate that acidic pH potentiates IgA-mediated Erk1/2 signaling in neutrophils, in line with the observed increase in LTB4 secretion.

## 4. Discussion

This study demonstrates that acidic pH, in combination with IgG- and IgA-mediated immunotherapy, consistently enhanced important antitumor functions of neutrophils. Across four different tumor cell line models, antibody-mediated cytotoxicity was amplified in acidic pH, especially upon IgA antibody treatment. Surprisingly, this occurred despite unaltered Fc receptor expression profiles and even reduced antibody binding in acidic pH. Live-cell imaging revealed that IgA antibodies induce potent neutrophil swarming and clustering around tumor cells, indicated by significantly faster movement speeds over short distances. These dynamics were so dominant that differences between pH conditions could not be determined. Interestingly, protumor functions such as ROS production and NET formation were strongly reduced under acidic conditions, even upon IgG or IgA exposure. Lastly, we observed a distinct neutrophil activation profile with IgA antibody treatment, particularly under acidic conditions, characterized by decreased neutrophil roundness/increased spreading and secretion of LTB4. Our findings highlight that the acidic TME drives functional adaptations in neutrophils that enhance their antitumor potential, especially when combined with IgA-based immunotherapies.

While our in vitro system allows for precise control over extracellular pH and effector-to-target ratios, it does not fully capture the complexity of the TME, including stromal components, vasculature, hypoxia, and various immunosuppressive signals, and the broader metabolic environment associated with tumor acidosis. In vivo, extracellular acidification occurs alongside alterations in lactate and glucose availability, oxygen tension, bicarbonate concentrations, and other metabolites, which may independently influence neutrophil function. Thus, while our approach allows us to specifically assess the effects of extracellular pH, it does not recapitulate the broader content of the acidified TME. However, direct testing of neutrophil-mediated ADCC under acidic conditions remains technically challenging in vivo. Systemic pH-modulating agents, like bicarbonate or proton pump inhibitors, buffer acidity rather than induce it, and can cause off-target effects [22]. Conversely, strategies to locally modulate pH are underdeveloped, as acid-releasing polymers or acid-labile hydrogels remain difficult to control within dynamic tumor tissues [23]. Monitoring intratumoral pH in real time is another barrier, as imaging methods like MRI, SPECT, and photoacoustics offer limited spatial resolution and carry risks of ionizing radiation [24,25]. Fluorescent pH sensors, while more sensitive, suffer from poor tissue penetration and photobleaching, limiting their use for deep-tumor pH assessment [26,27]. Given these limitations, our in vitro approach using primary human immune cells and complementary functional assays across different tumor models provides a tractable and mechanistically insightful platform. Future advances in pH-modulating biomaterials and imaging technologies may allow for more physiologically relevant in vivo validation of the mechanisms described here.

This study addresses an important gap in understanding how extracellular acidity influences antibody-mediated cytotoxicity by neutrophils in cancer. While prior research has focused mainly on spontaneous cytotoxicity by NK cells, we specifically evaluated both IgG- and IgA-mediated mechanisms, highlighting the unique and underexplored potential of IgA antibody treatment. Matched assessments of PBMC-mediated IgG killing, together with functional assays, live-cell imaging, and cytokine profiling, offer complementary insight into immune dynamics under acidic conditions. Importantly, the use of primary human immune cells, clinically approved antibodies, and multiple tumor models enhances the translational relevance of our findings. However, our use of neutrophils from healthy donors should be taken into consideration, as these cells may differ from neutrophils in the TME. Although we previously found comparable ADCC capacity between peripheral blood neutrophils from cancer patients and healthy donors [18], circulating neutrophils do not necessarily reflect those exposed to tumor-derived signals within the TME, including extracellular vesicles, that may alter neutrophil phenotype and function. Thus, the extent to which such tumor-associated changes influence neutrophil-mediated ADCC under acidic conditions remains to be determined.

While neutrophils rapidly respond to infection or injury by migrating to the affected site and generating ROS and degranulation [15], in the TME their excessive ROS and NET release can promote epithelial genetic instability, tumor proliferation, immune suppression, and metastasis through entrapment and dissemination of circulating tumor cells [28,29,30,31,32]. For example, neutrophils isolated from tumor-bearing mice can suppress CD4^+^ and CD8^+^ T cell proliferation, whereas neutrophils from wild-type mice cannot [33]. Our study, supported by existing research, demonstrates that acidic pH directly suppresses ROS production and alters NET formation [15,34]. This occurs because there is restricted glycolytic capacity, and enzymes like NADPH oxidase, essential for both processes, lose efficiency at low pH [15,35]. Therefore, even though neutrophils from these tumors are primed for protumor activity by their environment, the acidic conditions of the TME prevent these protumor mechanisms from fully unfolding. Although our study shows that acidic conditions suppressed ROS production and NET formation while enhancing antibody-dependent tumor cell killing, the broader consequences of this functional shift for therapeutic efficacy and inflammatory responses remain to be determined.

Tumors rely on glycolysis, generating excess H^+^ that is transported across the cell membrane [3,36], resulting in a extracellular pH gradient that can extend over several millimeters depending on vascular density and blood flow [37]. These gradients shape immune cell behavior by creating local pH nanodomains that influence adhesion, cytoskeletal remodeling, and directional migration [34,36,38]. While pH below 7.2 disrupts chemotactic steering in neutrophils, it does not impair their migratory velocity [34,37,38]. Our data confirm and extend these observations by showing that both directional movement and migration velocity of neutrophils are not impaired but rather enhanced under acidic conditions during IgG- or IgA-mediated immunotherapy. Notably, IgA promoted significantly faster migration but shorter displacement, suggesting it promotes more focused swarming toward tumor targets. LTB4 has been described to play a central role in neutrophil recruitment and activation and acts as a key chemotactic amplifier during neutrophil swarming, providing a mechanism for the increased killing observed in acidic pH [39,40]. These findings combined indicate that antibody opsonization can override pH-imposed chemotactic suppression, maintaining neutrophil navigation and function even in an acidic TME by increasing LTB4 secretion.

Strong chemoattractants like C5a, secreted at inflammatory sites, further enhance neutrophil migration, with LTB4 acting as an intermediary chemoattractant required for efficient chemotaxis toward C5a [41]. However, LTB4 production is typically reduced under acidic pH conditions following C5a stimulation [42]. In contrast, we observed that exposure to IgA in acidic pH caused increased LTB4 secretion. This likely reflects distinct signaling mechanisms: C5a acts through G-protein-coupled receptors, whereas IgA signals through FcαRI crosslinking. Our findings suggest that FcαRI-mediated signaling is enhanced in acidosis, similar to what has been described for FcγRI, the high affinity Fc gamma receptor for IgG, which also signals via the ITAM-containing FcR gamma chain [43]. Upon crosslinking of FcγRI with IgG-immune complex, ITAM-associated Syk kinases phosphorylate the LTB4 receptor LTB4R1 (also known as BLT1), which forms a FcγRI/LTB4R1 molecular complex that boosts phagocytosis, bacterial killing and LTB4 release [44]. A comparable mechanism may underlie the potentiation of FcαRI signaling under acidic conditions.

Neutrophils are inherently short-lived and undergo apoptosis within hours unless rescued by survival signals from their environment or activation state, such as cytokine stimulation (e.g., GM-CSF, CXCL8), Fc receptor engagement, or TME-associated stressors like hypoxia and acidosis [45,46,47]. Apoptotic pathways converge on caspase-3 activation [47,48,49], whereas survival is modulated through anti-apoptotic signaling pathways, including PI3K, JAK/STAT, and mitogen-activated protein kinase (MAPK) pathways such as Erk1/2 and p38. Notably, extracellular acidosis enhances Erk1/2 and p38 phosphorylation, preserving mitochondrial integrity and suppressing caspase-3 activity [50]. Consistent with this, our immunoblot analysis revealed earlier and stronger Erk activation in neutrophils stimulated with IgA-coated beads at low pH. Together with the increased LTB4 expression under acidic conditions, these findings suggest that Erk signaling and LTB4 production may contribute to the enhanced cytotoxic activity observed after prolonged exposure. However, whether Erk activation is sustained during the subsequent functional assay and directly contributes to the prolonged cytotoxic response remains to be determined.

## 5. Conclusions

While the acidic TME is generally immunosuppressive, our study reveals that under acidic conditions neutrophils retain and even enhance their antitumor capabilities when IgG and IgA antibodies are present. Importantly, this enhanced activity is accompanied by reduced ROS production and NET formation, suggesting a shift toward an effector phenotype with diminished protumor-associated functions.

Our findings identify neutrophils as uniquely resilient antibody effector cells in acidic tumor microenvironments and provide new insight into how extracellular acidity shapes antibody-dependent immune responses. Future studies should determine how additional features of the tumor microenvironment, including hypoxia, stromal interactions, and metabolic constraints, influence these responses and evaluate their relevance in physiologically representative models. Such studies will further define the role of neutrophil-engaging antibody strategies, including IgA-based immunotherapies, in solid tumors.

## Figures and Tables

**Table 1:** Overview of antibodies used in this study.

Antibody	Clone	Label	Company	Catalog Number
7-AAD			BD Pharmingen	559925
Annexin V		PE	BD Biosciences	556421
CD14	M5E2	FITC	BD	555397
CD14	HCD14	PerCP-Cy5.5	Biolegend	325622
CD16	3G8	PE	BD Pharmingen	555407
CD16	3G8	PE-Cy7	BD	557744
CD16	eBioCB16	APC	Affymetrix	17-0168
CD19	HIB19	APC	Biolegend	302212
CD20	2H7	APC-H7	BD Pharmingen	560734
CD3	OKT3	BV510	Biolegend	317332
CD32	FL18.26	FITC	BD Pharmingen	555448
CD56	NCAM 16.2	PE/Cy-7	BD	335826
CD56	NCAM HCD56	BV510	Sony Biotechnology	2191700
CD64	10.1	AF647	Sony Biotechnology	2125060
CD66b	G10F5	V450	BD	561649
CD89	A59	PE	BD Pharmingen	555686
CD89	A59	PerCP-Cy5.5	Biolegend	354109
MPO	4A4		Bio-Rad	0400-0002
MPO	MPO-7	FITC	Agilent	F071401-2
p-Erk1/2			Cell Signaling Technology	CST 9102S
β-actin	AC-74		Merck	A2228-100UL
IRDYE 680rd goat anti-mouse IgG			Li-Cor	LI 926-68070
IRDYE 800cw goat anti-rabbit IgG			Li-Cor	LI 926-32211

## Data Availability

All data is reported in this paper and any additional information required to reanalyze the data is available from the lead contact upon request.

## Supplementary Materials

The following supporting information can be downloaded at https://www.mdpi.com/article/10.3390/cells15181663/s1: Figure S1: Specific SKBR3 tumor cell lysis by purified NK cells, freshly isolated PBMCs or PMNs at pH 7.4 or 6.3; Figure S2: Immune cell composition, viability and FcR expression are marginally affected by low pH; Figure S3: Gating strategies of viability and Fc receptor expression; Figure S4: TrackMate images demonstrating single particle tracking during live imaging; Figure S5: Multiplex cytokine array on PBMCs shows reduced cytokine secretion in acidic pH. Videos S1–S6, related to Figure 4. Video S1: IgG pH 7.4; Video S2: IgG pH 6.3; Video S3: IgA pH7.4; Video S4: IgA pH 6.3; Video S5: No Ab pH 7.4; Video S6: No Ab pH 6.3.

## Abbreviations

The following abbreviations are used in this manuscript:

ADCC	Antibody-dependent cellular cytotoxicity
BLT1	LTB4 receptor 1
CFSE	Carboyfluorescein succinimidyl ester
^51^Cr	Chromium-51
CSF3	Granulocyte colony-stimulating factor, or G-CSF
CTV	Cell Trace Violet
EGFR	Epidermal growth factor receptor
Erk	Extracellular signal-regulated kinase
ELISA	Enzyme-linked immunosorbent assay
FcαRI	Fc alpha receptor: CD89
FcR	Fc receptor
FBS	Fetal bovine serum
G-CSF	Granulocyte colony-stimulating factor, or CSF3
GD2	Disialoganglioside 2
HER2	Human epidermal growth factor receptor 2
IFN	Interferon
ITAM	Immunoreceptor tyrosine-based activation motif
LTB4	Leukotriene B4
mAbs	Monoclonal antibody
MAPK	Mitogen-activated protein kinase
MFI	Mean fluorescence intensity
MPO	Myeloperoxidase
NET	Neutrophil extracellular trap
NK	Natural killer
PBMC	Peripheral blood mononuclear cell
PFA	Paraformaldehyde
PMA	Phorbol myristate acetate
PMN	Polymorphonuclear leukocyte
RBC	Red blood cell
RLU	Relative light unit
ROS	Reactive oygen species
SEM	Standard error of the mean
TANs	Tumor-associated neutrophils
TME	Tumor microenvironment
