# Supplementary material for: Tumor Acidosis Boosts Antibody-Driven Cytotoxic Potential of Neutrophils
Source: Cells. 2026 Sep 15;15(18):1663. doi: 10.3390/cells15181663 (PMC13605220; doi:10.3390/cells15181663)
Supplement: Supplementary file 1 [file cells-15-01663-s001.zip › Supplementary Figures.pdf]

Supplementary Figures

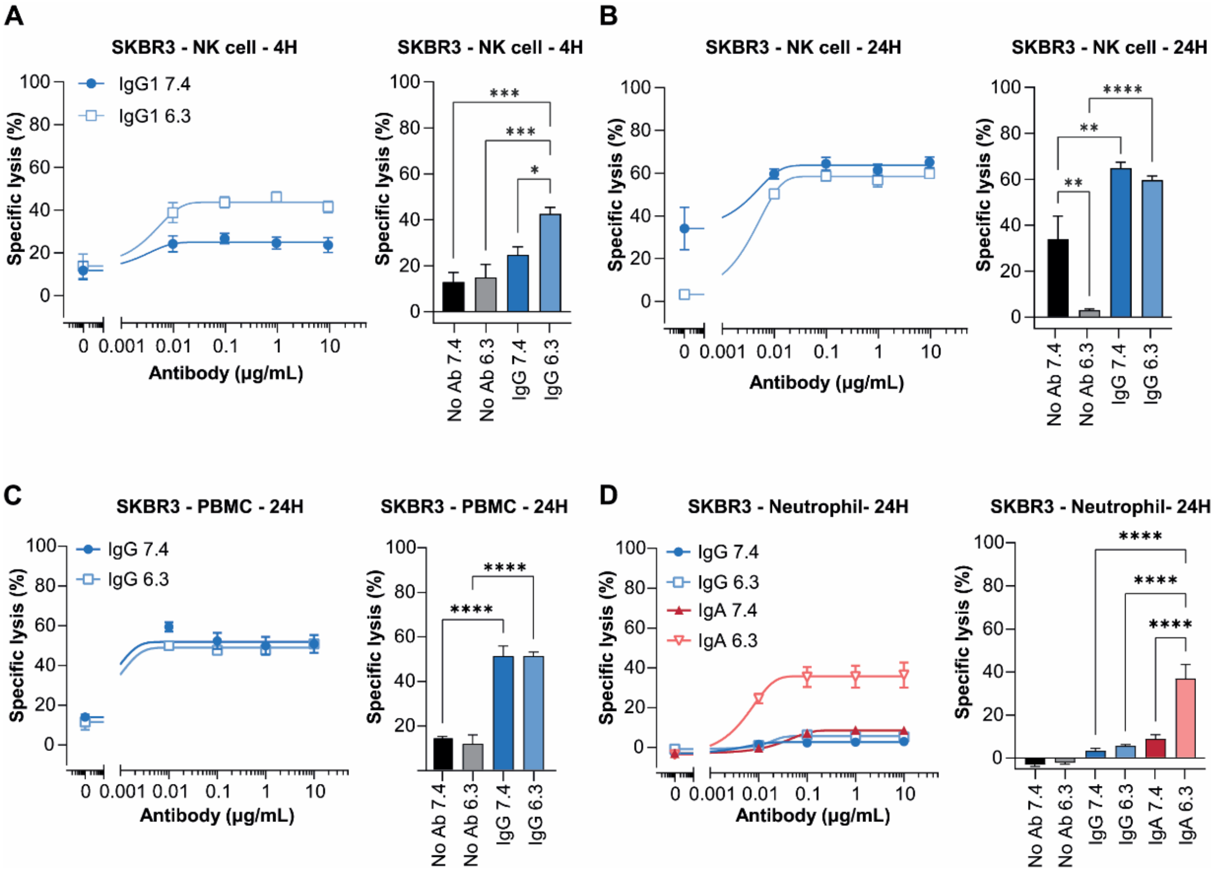

Figure S1. Specific SKBR3 tumor cell lysis by purified NK cells, freshly isolated PBMCs or PMNs at pH 7.4 or 6.3. Specific SKBR3 tumor cell lysis by **(A-B)** MACS CD14^{+} purified NK cells (E:T = 10:1), **(C)** freshly isolated PBMCs (E:T = 100:1) or **(D)** PMNs (E:T = 40:1) using a 4-hour 51Cr release assay at pH 7.4 or 6.3, or **(B-D)** after overnight culture in medium with their respective pH (i.e. 24 hours, including 20 hour overnight incubation). IgG1 and IgA anti-HER2 (trastuzumab) mAbs were used with SKBR3 target cells. Lines show mean with SEM for n=3-4 independent experiments with different healthy donors. Bar graphs show mean specific lysis of 3-4 experiments for IgG and IgA antibodies at 10 µg/mL. No Ab indicates condition without antibody. * p <0.05, ** p <0.01, *** p <0.001, **** p <0.0001, one-way ANOVA with Tukey's post hoc test.

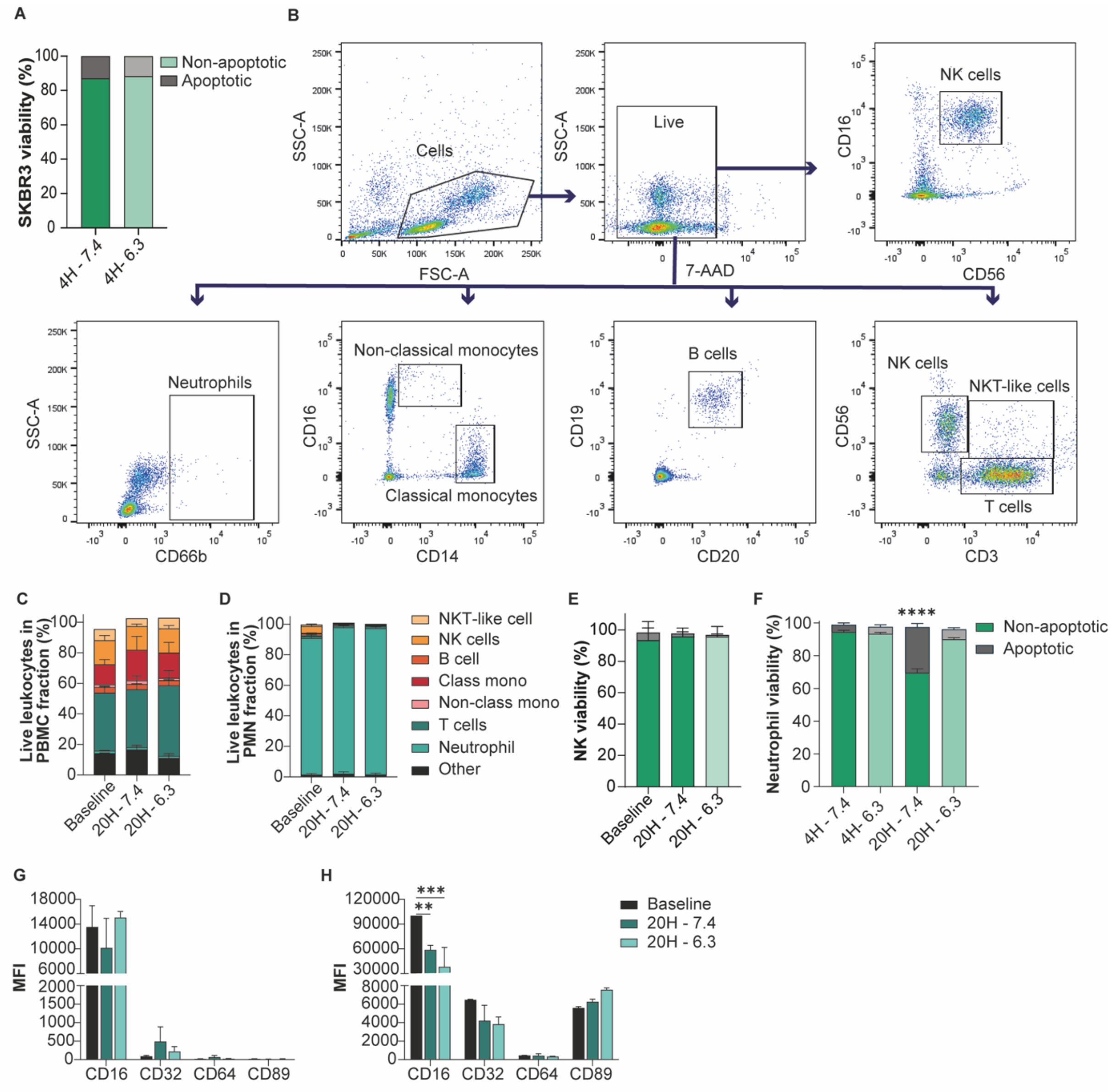

Figure S2. Immune cell composition, viability and FcR expression are marginally affected by low pH. (A) SKBR3 cells were cultured for 4 hours in RPMI medium with pH 7.4 or pH 6.3. Viability was determined using 7-AAD staining, and apoptosis was analyzed by flow cytometry with Annexin V. **(B)** The gating strategy is demonstrated for PBMC, with a similar approach applied for the neutrophil population. First, cells were gated on forward and side scatter, followed by a selection of live cells using 7-AAD. Subsequent gating identified immune cell subsets, such as NK cells (CD16+CD56+), neutrophils (CD66b+), classical (CD14+CD16-) and non-classical (CD14+CD16+) monocytes, B cells (CD19+CD20+), NKT-like cells (CD3+CD56+) and T cells (CD3+CD56-). **(C-D)** Immune cell compositions within the PBMC and neutrophil fraction. (**E-F**) PBMCs and neutrophils ($2x10^6$ cells/ml) were incubated for 20 hours in RPMI medium with pH 7.4 or pH 6.3. Viability was determined using 7-AAD staining. Apoptosis was analyzed by flow cytometry with Annexin V. 2-way ANOVA, with Tukey's post hoc test, **** p <0.0001 as compared to all other samples. (**G-H**) FcR expression on NK and PMN cells at baseline and after 20 hours in the respective pH. One-way ANOVA with Bonferroni post hoc test, ** p<0.01, *** p<0.001.

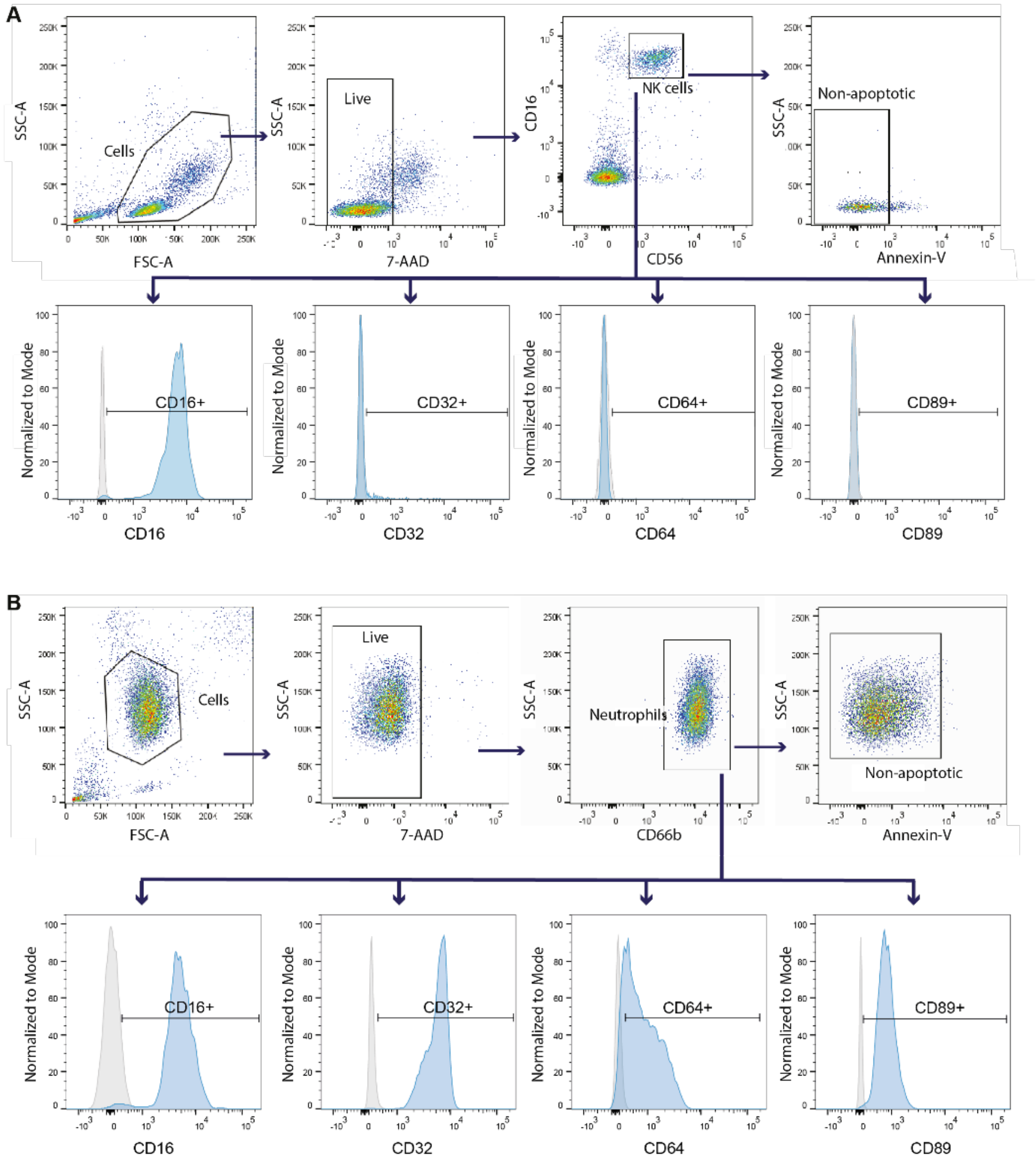

Figure S3. **Gating strategies of viability and Fc receptor expression.** Viability and Fc expression gating strategy for (**A**) NK cells and (**B**) neutrophils. First, cells were gated on forward and side scatter, followed by a selection of live cells using 7-AAD. Cell viability was further assessed by identifying non-apoptotic via Annexin-V. Fc receptor expression was determined within NK cell (CD16+CD56+) or neutrophil (CD66b+) populations. Grey histograms indicate the respective FMO (florescence minus one) controls, while blue histograms samples stained for Fc receptors.

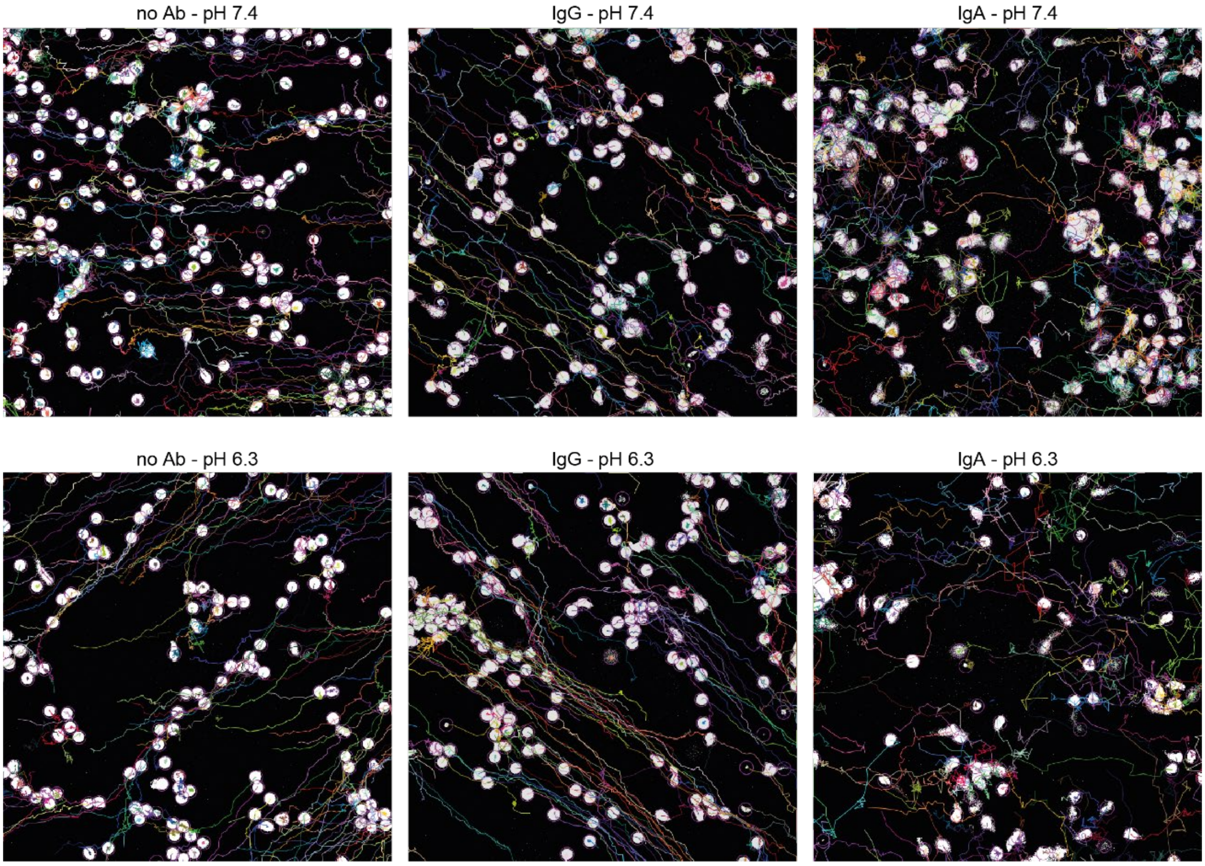

Figure S4. TrackMate images demonstrating single particle tracking during live imaging. Tracking of neutrophils with SKBR3 cells (E:T = 10:1) with IgG- and IgA-HER2 antibodies in live imaging over 30 minutes. Individual lines were used for mean square displacement and mean track speed calculations. A clear difference could be observed between IgG and IgA particle tracking. No clear differences were observed between pH conditions.

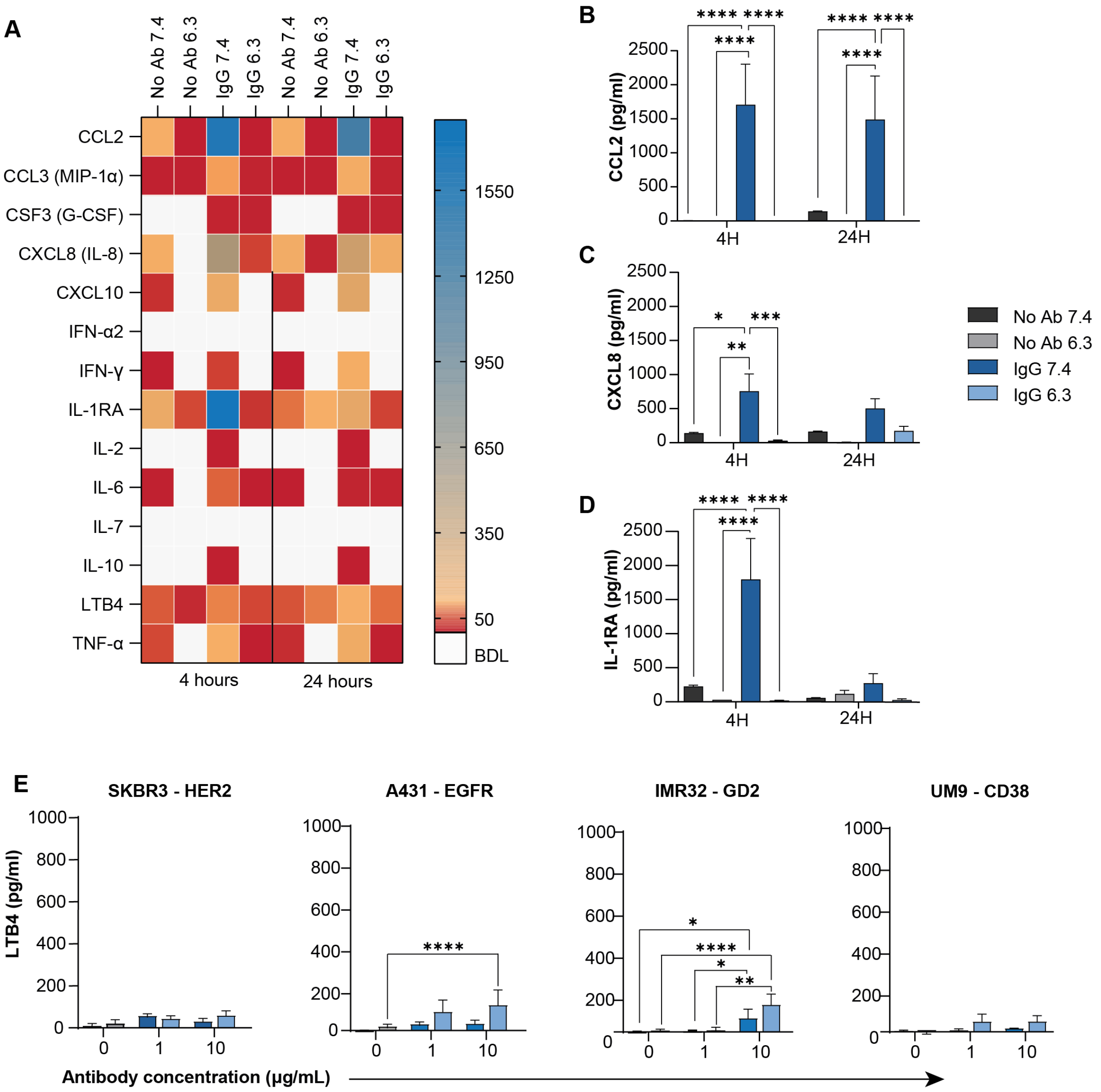

Figure S5. Multiplex cytokine array on PBMCs shows reduced cytokine secretion in acidic pH. (A) Multiplex cytokine array of SKBR3 cells with PBMC (IgG) in normal or low pH. Immune cells were freshly isolated from 3 healthy donors and either directly used in the assay for 4 hours, or cultured overnight in medium with their respective pH. Concentration in pg/ml. BDL = below detection limit. (**B-D**) Bar graphs of significantly altered cytokines excreted by PBMCs in multiplex. (**E**) LTB4 ELISAs of SKBR3, A431, IMR32, and UM9 cells. The same set-up was used as with the multiplex cytokine array, but 3 different healthy donors were used, measured in duplicate. Mean + SEM, Two-way ANOVA, Tukey's post hoc test. * $p < 0.05$, ** $p < 0.01$, *** $p < 0.001$, **** $p < 0.0001$.
